# Effect of Electric Field Distribution on the Heating Uniformity of a Model Ready-to-Eat Meal in Microwave-Assisted Thermal Sterilization Using the FDTD Method

**DOI:** 10.3390/foods10020311

**Published:** 2021-02-03

**Authors:** Yoon-Ki Hong, Roger Stanley, Juming Tang, Lan Bui, Amir Ghandi

**Affiliations:** 1 Department of Biological Systems Engineering, Washington State University, Pullman, WA 99164-6120, USA; jtang@wsu.edu; 2 Centre for Food Innovation, University of Tasmania, Launceston, TAS 7250, Australia; roger.stanley@utas.edu.au; 3 Defence Science and Technology Group, Scottsdale, TAS 7260, Australia; Lan.Bui@dst.defence.gov.au (L.B.); Amir.Ghandi@dst.defence.gov.au (A.G.)

**Keywords:** microwave heating, sterilization, FDTD numerical simulation, electric field distribution, heating pattern, heating uniformity

## Abstract

Microwave assisted thermal sterilization (MATS) is a novel microwave technology currently used in the commercial production of ready-to-eat meals. It combines surface heating of high-temperature circulation water with internal microwave heating in cavities. The heating pattern inside the food packages in a MATS process depends heavily on the electric field distribution formed by microwaves from the top and bottom windows of the microwave heating cavities. The purpose of this research was to study the effect of the electric field on 922 MHz microwave heating of ready-to-eat meals as they moved through the microwave chamber of a pilot-scale MATS system using the finite-difference time-domain (FDTD) method. A three-dimensional numerical simulation model was developed as a digital twin of the MATS process of food moving through the microwave chamber. The simulation showed that the electric field intensity of the MATS microwave cavity was greatest on the surface and side edge of the cavity and of the food. There was a strong similarity of the experimental heating pattern with that of the electric field distribution simulated by a computer model. The digital twin modeling approach can be used to design options for improving the heating uniformity and throughput of ready-to-eat meals in MATS industrial systems.

## 1. Introduction

Microwave-assisted thermal sterilization (MATS) is an advanced thermal processing method that has been applied to produce pre-packaged, shelf-stable food products [1]. Volumetric microwave heating in the system can expeditiously increase the temperature of food sealed in packages, thereby providing shorter heating times and less quality degradation [2]. The system uses 915 or 922 ± 15 MHz to provide greater microwave penetration depth and more uniform heating than the 2450 ± 50 MHz multi-mode cavities used for domestic microwave ovens or microwave drying systems [1]. Additionally, the design provides relatively predictable heating patterns during the continuous heating process with conveyance of the sealed food trays [3].

For microwave heating, various prior approaches have been attempted to improve the electric field uniformity during the heating of the food. These include moving the food through the electric field using conveyor belts or turntables, using mode stirrers, rotary radiation, or changing the mode, frequency, and dimensions [4,5,6]. However, overheating at the edges of food can occur with high rates of heating due to the impedance difference between high-moisture foods and air. This has been a challenge to improving the heating uniformity for domestic and industrial purposes [1]. To achieve rapid heating without hot spotting causing thermal runaway and burning of the foods, the MATS system uses pressurized immersion in water. The requirement for commercial MATS success is, therefore, to be able to get high throughput and efficient use of the capital investment, which is much higher for pressurized microwave retorting than for non-pressurized microwave heating of foods in the air.

The commercial MATS system was designed based on the research MATS system at Washington State University (WSU) [7,8,9,10,11]. Commercial units are being used in several companies and institutions to sterilize various types of products [12]. While the pilot-scale MATS system at WSU has been validated by extensive research on heating uniformity [7,8,11,13,14], there still exist opportunities to improve the design of commercial MATS systems for improved throughput and product quality. Extensive testing at the pilot scale is necessary to ensure that throughput and energy efficiency are optimized for the industrial-scale system. According to Luan et al. [7], the dimensions of each part in the system affect the electric field distribution during microwave heating. In turn, the electric field distribution in the system affects the overall heating patterns and uniformity. Therefore, understanding the electric field distribution in the system as food packages move through the heating chamber is necessary to improve heating uniformity and optimize the processing of different products and packages.

Numerical simulation is a cost-effective and convenient tool to determine the electric field distribution in a cavity and a product. Numerical simulation has been widely applied not only in the food industry but also in various biological processing applications to estimate heating patterns and electric field distributions [15]. The determination of hot and cold spots in sealed food packages is an essential step in optimizing the thermal processing conditions for producing safe foods of premium quality. However, the simulation of heating patterns in microwave heating is much more complicated than that for conventional thermal processing using steam or hot water since Maxwell’s equations for electromagnetic field need to be considered along with the general heat transfer equations for microwave heating. In a MATS process, the food packages under high pressure are heated by microwaves while immersed in hot circulating water, followed by rapid cooling in cold water to stop further thermal degradation reactions. The process can therefore achieve thermal sterilization in a shorter period of time compared to conventional batch retort heating. However, the heating pattern in microwaves may be altered by the dimensions of products and material properties [16]. Three-dimensional (3-D) computer simulations, using the finite-difference time-domain (FDTD) method, have previously been utilized to simulate electric fields and heating patterns in the MATS system at WSU. Numerous studies have been conducted to determine the effect of various factors such as generator power and frequency, the dielectric properties of water, temperature sensor, and carrier design on the heating pattern in the MATS system [7,8,9,10].

The dimensions of the system are one of the critical factors affecting the electric field distribution and heating patterns in the microwave cavity [4]. Thus, dimension changes made in the microwave cavities may alter the electric field distribution in a heating cavity and consequently change the heating patterns in food packages [7,14]. On the other hand, the dielectric properties of the foods, in turn, are affected by the temperature, with greater microwave energy loss to the food at higher temperatures. This interaction between the electric field and the dielectric microwave properties of the foods governs the heating pattern of a food product during microwave heating [7].

Previous research determined the electric field distribution on the pilot-scale MATS at WSU. However, the system dimensions, such as horn design and cavity size, are different from those for the MATS-B system [4,5], which was designed to replicate one microwave cavity of a full commercial production system that has multiple microwave cavities in series. These scale-up changes affect the overall electric fields, along with their impacts on heating uniformity. For this reason, in this research, we aimed to use computer simulation to understand the effects of dimension changes in the commercial MATS system on the predicted electric field distribution during movement of the food through the microwave chamber, and the consequential heating patterns. In addition, the heating uniformity can change depending on the position of food trays in the transport carrier as it moves through the cavity. We therefore also simulated the effects of movements of the trays through the electric field. A uniform simulated model food, in the form of a starch gel, was used to allow control over the dielectric properties and eliminate thermal convection within food packages. This facilitated the calculation of the interactions between the electric field and the microwave properties.

The validity of the projected heating pattern then needed to be assessed by comparison with experimental measurements on the MATS-B. This was done using a chemical-marker-based model food and a computer vision system. The availability of a predictive simulation model could, in the future, be applied to optimize variables in microwave-assisted thermal processing technology to achieve better rapid heating uniformity, process efficiency, and improved product quality.

## 2. Materials and Methods

### 2.1. 3D Numerical Simulation

The computer simulation model for MATS was built using Quickwave software (QWED Version 7.5c, Warsaw, Poland). The software uses the conformal FDTD algorithm based on Maxwell’s equations [17]:

(1) $$\oint_{c}^{\;}E\cdot dl=-\frac{\partial }{\partial t}\int \int_{s}^{\;}\mu H\cdot ds,$$

(2) $$\oint_{c}^{\;}H\cdot dl=-\frac{\partial }{\partial t}\iint_{s}^{\;}\varepsilon E\cdot ds+\iint_{s}^{\;}j_{c}\cdot ds,$$

(3) $${∯}_{s}^{\;}E\cdot ds=\frac{Q_{e}}{\varepsilon }\;,$$

(4) $${∯}_{s}^{\;}H\cdot ds=0,$$

where E is the electric field intensity (V/m), μ is the magnetic permeability (H/m), H is the magnetic field intensity (A/m), ε is the electric permittivity (F/m), $j_{c}$ is the conduction electric current density (A/m^2^), and $Q_{e}$ is the total free electric charge enclosed by surface S. Subscript ${\;}_{C}$ on $j_{c}$ indicates the conduction current density σE (C). The dissipated power in a material is expressed as [18]

(5) $$P=2\pi f\varepsilon_{0}\varepsilon_{r}^{\prime \prime }{\left|E\right|}^{2},$$

where f is the frequency (Hz), $\varepsilon_{0}$ is the permittivity of free space $\left(8.85\times 10^{-12}\;F/m\right)$, and $\varepsilon_{r}^{\prime \prime }$ is the dielectric loss factor. In this study, solid-type model food with no thermal convection was used to determine the heating patterns during processing. The General Heat Transfer equation is expressed as

(6) $$\nabla^{2}T=\frac{\rho C_{p}}{k}\frac{\partial T}{\partial t}\;,$$

where *T* is the food temperature(°C), ρ is the density (kg/m^3^), $C_{p}$ is the heat capacity (J/(kg·°C)), k is the thermal conductivity (W/(m·°C)), and *t* is time (s). The heat flux *q* (W/m^2^) at the boundary between food and water was stated as

(7) $$q=h\left(T-T_{s}\right),$$

where *h* is the convective heat transfer coefficient (W/(m^2^·°C)), and $T_{s}$ is the temperature of circulating water in the MATS system. h was set to 190 W/(m^2^·°C) based on a calculation using dimensionless numbers and process conditions [19].

**Figure 1:**
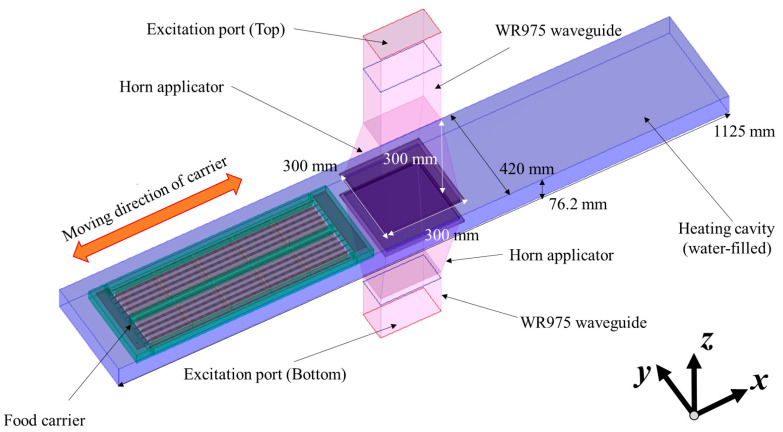
3D geometry of the MATS system with food transportation carrier in Quickwave software.

The geometries of the model in the microwave heating section were built based on a MATS-B pilot system (https://www.915labs.com/systems (accessed on 15 January 2021)) installed at the Defence Science and Technology Group facility, Scottsdale, Tasmania, Australia (Figure 1). The details of the basic concept of the MATS system are as described in Luan et al. [7]. The system has a microwave generator (Thermex Thermatron TM100, Louisville, KY, USA) connected to a waveguide that splits to feed two microwave horn applicators located at the top and bottom of the heating cavity. Sealed trays of food are placed in a carrier and conveyed by a chain drive through the pressurized chamber filled with circulating pressurized hot water. The radiated microwave power was set to 8.5 kW based on the real MATS system. The dimensions, and the thermal and microwave properties of the food and the carrier, were used to predict the electric field and heating pattern of the food in the trays. The body of the carrier was made of polytetrafluoroethylene (PTFE)-based plastic, and the lid of the carrier was stainless steel with a grill of longitudinal metal bars to hold the trays in place. The properties of the plastic were obtained from the literature [20,21]. The arrangements of the microwave cavity and the food carrier are shown in Figure 1. The mesh size of the simulation model was set based on the rule of more than ten cells per wavelength [22]. The total cell number was 10,316,850, and it took 72 h per simulation run. In the previous studies, 16–32 steps were proven to be adequate in simulations for food packages moving through four microwave cavities [8,10,23], while 44 steps were used in heating pattern validation in the current research. The simulation model was used to determine the electric field distribution of the cavity in the MATS system, with and without carrier trays. The thickness of the sample was set to 26 mm based on the maximum depth of the 430 g tray (loaded weight, dimensions 152 × 108 × 32 mm). The QuickWave Basic Heating Module (BHM) add-in was applied to ascertain the heating patterns of food products during simulated movement of the carrier with the trays of food through the heating cavity between the microwave horns. The simulation considered convection heat transfer at the interface between water and foods. In solid or gelled foods, thermal conduction is the only mechanism for internal thermal transport. The detailed simulation parameters and properties are described in Table 1.

### 2.2. Experimental Validation

#### 2.2.1. Model Food Preparation

Model foods for MATS research have been formulated based on whey gels and used for over a decade for color mapping of heating patterns [24,25,26]. However, purified whey protein is expensive for larger-scale research, and the preparation method is time-consuming. The use of a starch-based model food was investigated in this study with the aim of avoiding the cost, sourcing, and preparation difficulties associated with the whey gel model food.

Rice flour and tapioca starch have been used widely in Asian countries to prepare starch-based dishes with a flexible gel-like structure. A rice-based model food developed by Auksornsri et al. [27] for MATS processing was trialed, but it was too firm, dried out quickly, and did not cut cleanly. A new formulation based on rice flour, tapioca starch, potato flakes, and xanthan gum was developed to achieve the properties needed for this study. This starch-gel model food produced a flexible, strong gel structure that was easy to handle both prior to and post MATS processing. It also provided a clean, smooth surface when cut, facilitating preparation for photographing and image analysis. In addition, it can be used for other purposes, such as the addition of flavors, vitamins, or spores for food quality or microbiological studies. Fructose or ribose was added to the level required for the development of Maillard browning at sterilization temperatures to distinguish variations in thermal exposure.

Specifically, the model food was a combination of rice flour (400 g, Erawan brand, product code 55104001170, Nakhon Phantom, Thailand), tapioca starch (100 g, Erawan brand, product code 55104001163, Bangkok, Thailand), dehydrated potato flakes (300 g, AGRI, product code W062982, Warden, WA, USA), fructose (40 g, Imported from Finland by Natures Works, Launceston, TAS, Australia), water (1900 g, ~35 °C), 0.5% xanthan gum suspension (100 g, imported from China by Natures Works, Launceston, TAS, Australia), and non-iodized salt (20 g, Salpak Pty Ltd., Seven Hills, NSW, Australia) for the added-salt model food. The mixture was prepared using a rotary bowl mixer (Kenwood Chef XL Titanium, Havant, United Kingdom) with a flexible beater. Water was added to the mixing bowl, then all dry ingredients were added gradually while mixing at low speed until a smooth paste with no lumps was achieved. Xanthan gum solution (0.5%) was then added to the mixture and mixed well. The model food mixture (430 g each tray) was transferred to rectangular multi-laminate polypropylene (PP), polyethylene (PET), and ethylene vinyl alcohol (EVOH) barrier retort food trays (tray size 152 × 108 × 32 mm, Printpak, Atlanta, GA, USA). The filled food trays were placed into baking trays; each was covered with an empty food tray and water was added to the base of the baking trays to a depth of approximately 1 cm, prior to placing them in a combi-oven (Unox Chef Top, model XEVC-0711-EFR, Cadoneghe, Italy) for steaming to set the model food mixture. Steaming took place at 90 °C for 40 min, fan setting 1, with a 30% steam level.

Trays of the model food, with and without added salt, were prepared fresh for each MATS run. Duplicate MATS runs were performed for the control model food (no added salt, *n* = 6) and the added-salt model food (*n* = 6). Control trays of the model food were also prepared (with and without salt) but not processed through the MATS system.

#### 2.2.2. MATS Processing Conditions

Processing was conducted using a MATS-B system (915 Labs Inc., Denver, CO, USA) operating at a peak of 922 MHz. The MATS was located in the food research pilot plant of Defence Science and Technology, Scottsdale, Tasmania, Australia. Variations in the peak frequency from 915 MHz within the industrial, scientific, and medical (ISM) band (915 ± 15 MHz) do not significantly alter the heating patterns in 915 MHz cavities in a MATS system [10]. Microwaves propagated from the generator divide at a tee split of the waveguides and entered from the top and bottom of the cavity to set up resonating standing wave patterns. An isolator was used to absorb reflected energy from the cavity to avoid microwave signal coupling and safeguard the generator. The heating system consists of three coupled pressurized processing sections: pre-heat, microwave, and cooling. Pressure (50 psi = 345 kPa) was applied to the chamber to prevent the food package from bursting during the microwave heating. In addition, the glass-reinforced PTFE carrier had longitudinal stainless steel bars top and bottom to restrain the sealed packages of model food during microwave exposure in the water-filled processing chambers. The carrier moved through the three chambers at constant speed via a chain drive mechanism. In operation, the circulation water was pumped through an external circulation system and the water temperature (124 °C) was maintained by heat exchangers outside of the microwave system. The microwaves were confined in the enclosed metal pressure chambers of the MATS system.

The tray carrier accommodated 10 trays per run. The tray locations of interest for this study were the two trays at each of the leading and trailing ends and in the middle of the carrier. Sets of six sealed model food trays were placed in the carrier at the locations just described. Four dummy trays containing the model food were inserted between the leading and middle trays (×2) and the middle and trailing trays (×2) of the carrier. The carrier stainless steel grill lid was closed and secured with cable ties prior to loading it in the MATS system (Figure 1). It was first loaded into the pre-heating section to pre-heat the products to an even temperature (85 °C for 25 min). The carrier was then transferred through a pressure gate to the microwave section for further heating and sterilization, prior to moving into the cooling section through another pressure gate for cooling before removal from the system. While in the microwave vessel, the carrier moved forward and backward through the 922 MHz microwave applicator (Figure 1) for a total of four passes, then was held for a set period of time and temperature for sterilization without further microwave application, as detailed in Table 2 for sterilization process conditions.

Following processing, the trays were photographed, as described in Section 2.2.4, to determine the heating patterns using a false-color computer vision method [28]. Water of less than 10 µS/cm conductivity from a reverse-osmosis system was used as the circulating water for the MATS in operation. The temperature of the circulating water was controlled to remain at 121 °C through a heat exchanger outside the MATS unit to give the temperature as specified in Table 2.

#### 2.2.3. Heating Pattern Analysis Using a Computer Vision Technique

On completion of the steaming process to set the gel, the model food trays were removed from the combi-oven, and the surface of the trays was photographed using a digital camera (Canon EOS 5D Mark IV (WG), Tokyo, Japan), with a 50 mm F1.4 DG HSM lens, Sigma Corporation, Kawasaki, Japan) prior to vacuum sealing the trays (SuperSealer, model REI-70, Tullamarine, VIC, Australia) with a retort barrier film (Printpack, Atlanta, GA, USA), ready for MATS processing (Section 2.2.2). Trays were sealed at 176 °C for 15 s, with nitrogen flushing and −0.065 MPa vacuum.

After MATS processing, the temperature of the trays was around room temperature (20 °C); the retort barrier film was removed and the processed model food samples were again photographed for heating patterns by color mapping of the Maillard browning reaction [28]. All model food samples (controls with and without added salt, and treatment samples with and without added salt) were photographed to capture the heating patterns as follows: the model food was sliced horizontally along the middle to photograph the middle surface; then the two halves were put back together, without deforming the model food structure; and, finally, vertical sections were cut to form three equal portions for photographic recording of the cross-sectional heating patterns.

#### 2.2.4. Dielectric and Thermal Property Measurement

The dielectric and thermal properties of the model foods were measured (*n* = 6) using a Network Analyzer (Keysight E5071C, ENA series, Penang, Malaysia) with a coaxial dielectric probe (Agilent Technologies, Santa Clara, CA, USA) and a thermal analyzer (Tempos, Meter Group, Inc. USA, Pullman, WA, USA). The measured temperature range for dielectric properties was 30 to 130 °C, and that for thermal conductivity properties was 30 to 90 °C.

## 3. Results and Discussion

### 3.1. Electric Field Distribution of the Empty Microwave Cavity

**Figure 2:**
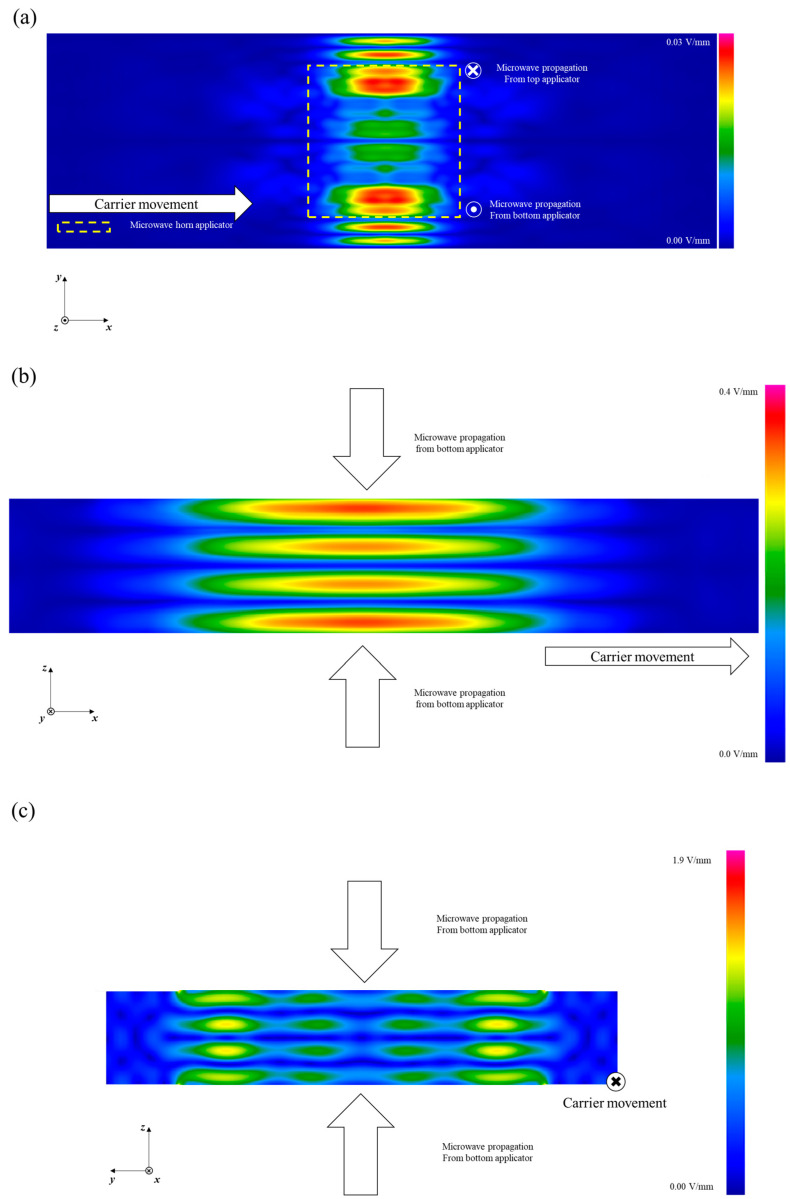
Time average electric field in the empty cavity of the MATS system. (**a**) *x–y* plane, (**b**) *x-z* plane, and (**c**) *y–z* plane (scales are adjusted for better electric field visualization).

The electric field distribution in the empty heating cavity (prior to the entry of the food packages) was analyzed using the computer simulation model. The average electric field intensity on the *x–y* plane, in the empty cavity of the MATS system, gives anti-nodes and nodes symmetrically distributed along the *y*-axis at the center of the cavity (Figure 2a). This is because of the changes from single-mode to two modes in the cavity along the length of the *y*-axis. The width of the cavity, greater than the width supported by single-mode, causes the electric field to be divided into multi-modes in the *y*-direction, as described by Pathak et al. [13] and is the reason why the highest electric field intensity was located at the end sides of the horn applicator (Figure 2a). This can be compared with the electric field distribution in the pilot MATS system at WSU, which showed a center-focused electric field distribution with a narrower heating cavity [14]. High-field-intensity zones were observed at the central plane in the *y*-direction in the system at WSU [7,8,10]. In contrast to those in the current research, the high-field-intensity zones were more focused on the center of the cavity.

Microwaves in the system are propagated towards the *z*-direction from the top and the bottom. This causes standing wave patterns in the heating cavity. On the *x–z* plane, four anti-nodes and three nodes with standing wave patterns were predicted to be in the heating cavity (Figure 2b). Three nodes and four anti-nodes were detected in the heating cavity on the *x–z* plane. This standing wave pattern is similar to the pattern of 180° phase difference between top and bottom microwaves reported by Luan et al. [7]. When two transverse waves enter the top and the bottom of the cavity with a 180° phase difference, the waves cancel each other, forming a node of electric field minimum located at the center of the cavity. In contrast, when there is a 0° phase difference for two transverse waves encountering each other in the cavity, the anti-node of maximum electric field intensity is positioned at the center of cavity where the center depth of the food occurs. The electric field intensity at the center of cavity should therefore result in better volumetric microwave heating when food samples move through the center of the cavity [7]. On the *y–z* plane, the anti-node positions are located at the side of the cavity (Figure 2c). A side- and surface-focused electric field distribution could aggravate non-uniform heating during processing.

### 3.2. Electric Field Distribution of the Loaded Microwave Cavity

**Figure 3:**
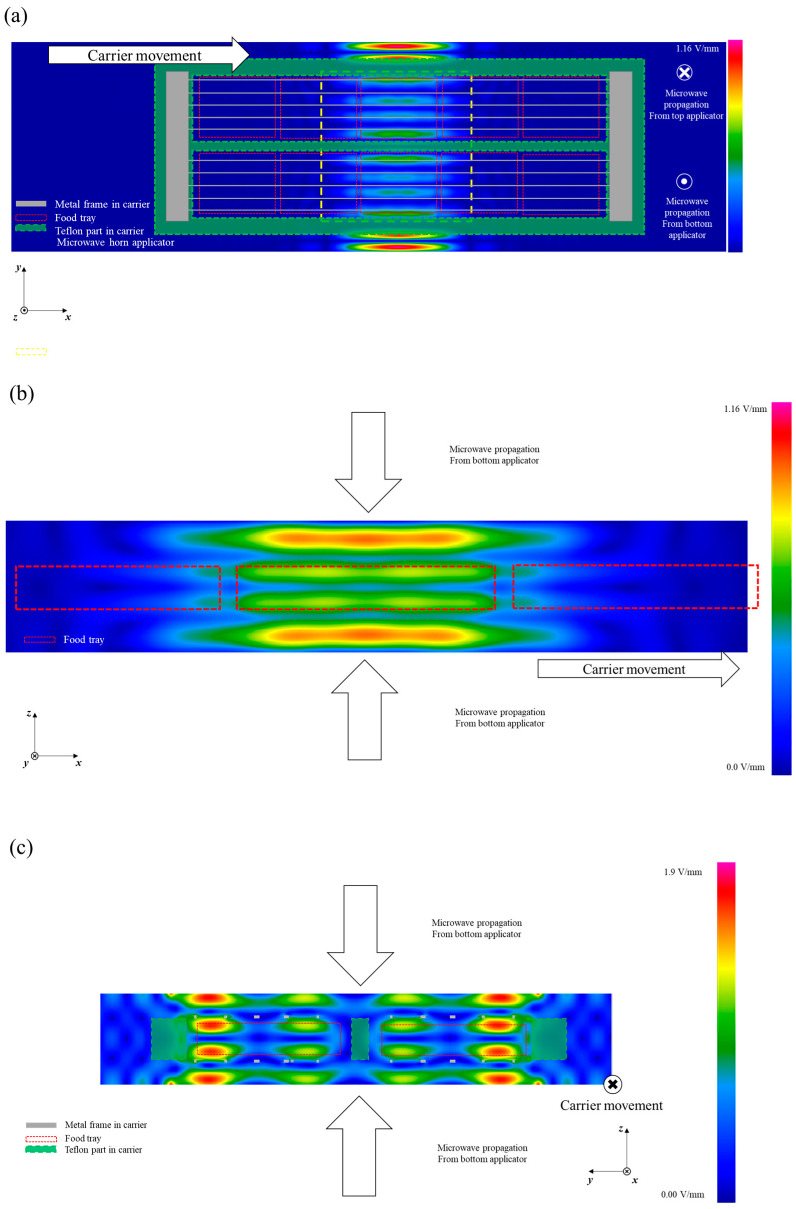
Time average electric field in the cavity of the MATS system with a food transport carrier. (**a**) *x–y* plane, (**b**) *x–z* plane, and (**c**) *y–z* plane (scales are adjusted for better electric field visualization).

The electric field distribution in the cavity can be changed by the presence of a food carrier loaded with ten food trays [8]. Figure 3 shows the electric field distribution in the cavity when the food transport carrier was located at the center of the cavity. As shown in Figure 3a, higher energy intensity was presented at the side edge of the food and the carrier as compared to food at the center of the cavity on the middle layer of the cavity (*x–y* plane). On the *x–z* plane, the standing wave patterns with three nodes and four anti-nodes were similarly presented as for the electric field without the carrier (Figure 3b). However, a more intense electric field was predicted in the heating cavity. The electric field distributions on the *x–z* and *y–z* planes were also changed by the presence of the food transport carrier, but the overall patterns were similar to the pattern of the empty cavity with regard to their node and anti-node positions. Based on the time average electric field distribution, the surface (top and bottom) and outside (close to the edge of the carrier) would achieve a faster heating rate due to microwave energy compared to the inner parts of the food packages. Jain et al. [8] reported that metal-framed food transport carriers did not change the general electric field distribution but did change the electric field intensity.

**Figure 4:**
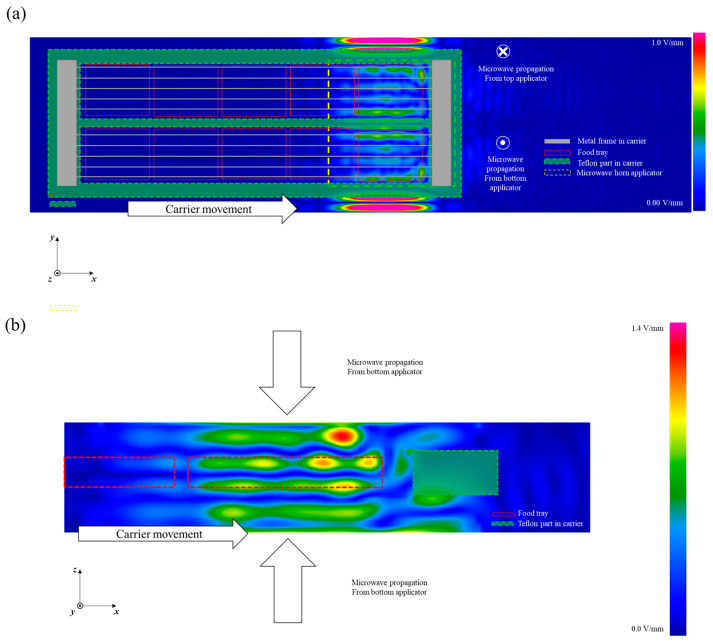
Time average electric field in the cavity of the MATS system when the edge of the food transport carrier is located at the center of the cavity. (**a**) *x–y* plane, (**b**) *x–z* plane (scales are adjusted for better electric field visualization).

The time average electric field for when the edge of the food transport carrier was located at the center of the cavity is shown in Figure 4. The electric field inside the food at the edge of the carrier was more focused on the side edge near the end of the carrier compared to the electric field inside the food located at the center of the food carrier. In general, pure water is a much lower microwave energy loss medium compared with food materials and does not significantly absorb and convert microwave energy to thermal energy. The higher electric field intensity predicted at the side edge of the carrier would cause a leading-edge heating effect for the foods at the side of the carrier (Figure 4a).

The predicted standing wave patterns along the *z*-direction were similar with and without the food located at the center of carrier, having three nodes and four anti-nodes in the cavity (Figure 4b). However, the electric field was not symmetrical with respect to the center of the cavity in the *z*-direction due to the metal plate securing the grill that was placed on the top edge of the transport carrier. To improve the heating uniformity of the system, various techniques were investigated in previous research, such as changing frequencies, or changing the shape and size of samples [29,30]. Also, Zhu et al. [6] improved the heating uniformity in the microwave heating cavity (2450 MHz) using a rotary radiation structure. Rotating metal patches or different shapes of stirrers were applied in stir mode, which improved the temperature difference between hot and cold spots in a 2450 MHz multi-mode microwave cavity [6,31]. These studies tested changes in mode (various patterns) during microwave heating to obtain better heating uniformity; however, these can cause complicated and unpredictable heating patterns during the processing of ready-to-eat meals [1]. In this regard, the non-uniform edge heating at the side edge of food products could be altered, in the future, by adjusting the placement of the metal plates as a practical solution [8].

### 3.3. Actual Heating Pattern Analysis with Model Food

The model food was developed to help us more readily determine the heating patterns of microwave-assisted thermal processing systems using the Maillard browning reaction [27,32,33,34]. It has been shown that the Maillard reaction in model foods containing M-2 (4-hydroxy-5-methyl-3(2H)-furanone) chemical marker precursors follows a first-order reaction and that the corresponding color changes in the model foods reliably reflect the intensity of thermal inactivation of *Clostridium botulinum* spores [28,34,35,36]. In contrast, in conventional retort heating methods (e.g., using only pressurized hot water and steam), the cold spots of microwave heating are not always located at the geometrical center or near the center because the heating pattern is related to the electric field distribution. The MATS systems use a combined microwave and hot water heating mechanism. Therefore, although we can get an overall electric field distribution and heating patterns from computer simulations, validation of the actual heating patterns is very important for improving the system design.

**Figure 5:**
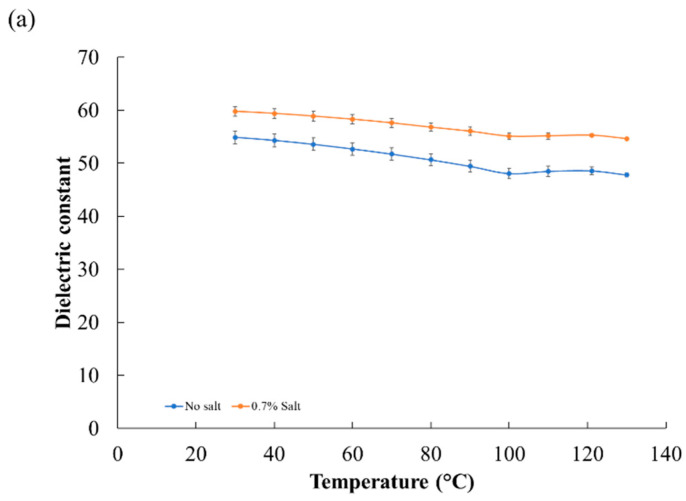

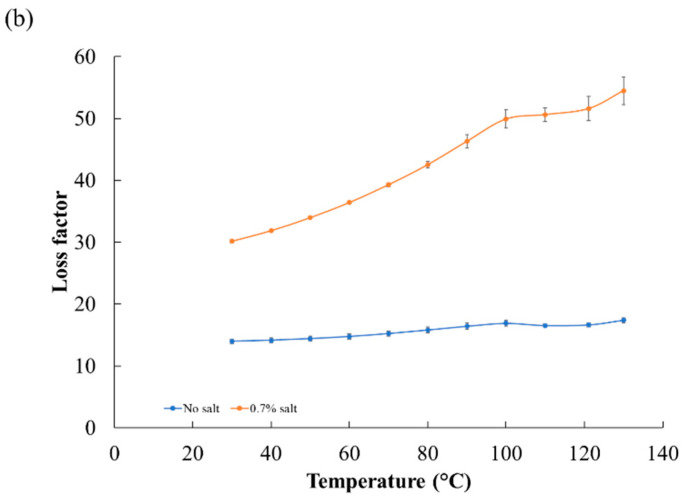
Dielectric properties of potato–rice-based model foods: (**a**) dielectric constant and (**b**) loss factor.

The dielectric properties of the model foods are shown in Figure 5. The dielectric constant and loss factor are, respectively, defined as the ability to store electric energy and the conversion of microwave energy to thermal energy. The dielectric constant increased with salt content but decreased with increasing temperature. The changes in salt level most significantly affected the loss factor. The loss factor of the model food without added salt did not increase much with a temperature increase from 30 to 130 °C, while the loss factor of the 0.7%-added-salt model food dramatically increased with temperature. The effect of salt on dielectric properties was reported in previous research, where it was shown to change the dielectric loss factor significantly [27,37,38]. The overall trends in dielectric properties with temperature and salt level are similar to those for the rice-based model food reported by Auksornsri et al. [27].

**Figure 6:**
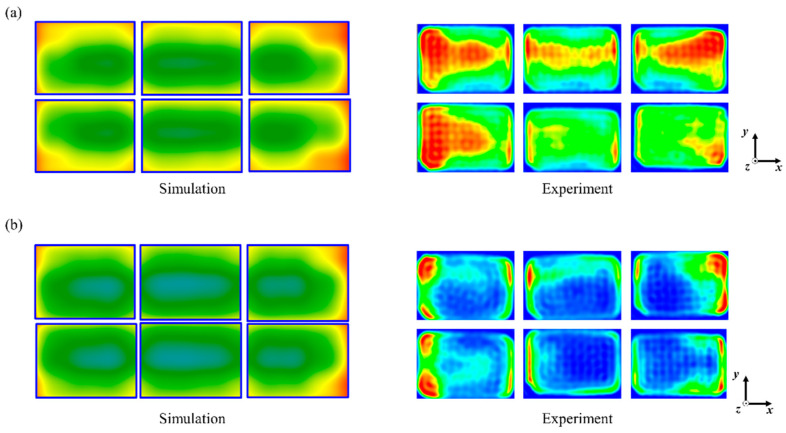
Comparisons of heating patterns on the middle layer of model foods: (**a**) 0.0%-added-salt and (**b**) 0.7%-added-salt model foods.

Experimental validation of the simulation results was undertaken using the MATS system, noting that while the cavity dimensions of the MATS-B were replicated in the simulation, the 6° angle tilt of the horns from vertical and the internal partitions in the horn could not be simulated due to computer processing power limitations. The heating patterns were determined using the computer vision method [28]. The experimental heating pattern is shown in Figure 6 using relative color changes in model foods indicated by a red-to-blue color scale based on the formation of brown color by the Maillard chemical marker. The heating patterns of the model foods determined by simulation and experiment are compared.

The heating patterns in the middle layer of the food for both the simulation and the experimental outcome showed similar locations of cold and hot spots. The trays located at the sides of the carrier received more thermal energy than the trays at the center of the transport carrier for the 0.0% and 0.7% salt model foods, although there was a color intensity difference. Furthermore, the trays located at the side ends of the carrier presented much higher thermal intensity in both the simulation and experiment results. The more intense energy at the side edge of the food tray is considered to result in edge heating during microwave heating. This is similar to the electric field distribution in the food (Figure 3 and Figure 4). In addition, the results can be explained by the microwave penetration depth as determined by the dielectric properties of each model food. The penetration depths of microwaves into model foods with and without 0.7% salt were 25 and 8 mm, respectively. Higher loss factors cause lower microwave energy penetration into the food. Jain et al. [16] and Hong et al. [39] reported a similar trend that an increased loss factor due to a high salt level in food resulted in greater energy absorption at the edge than at the center of foods such as pea, mashed potato, and rice in a pilot-scale MATS system and microwave-assisted pasteurization system (MAPS).

**Figure 7:**
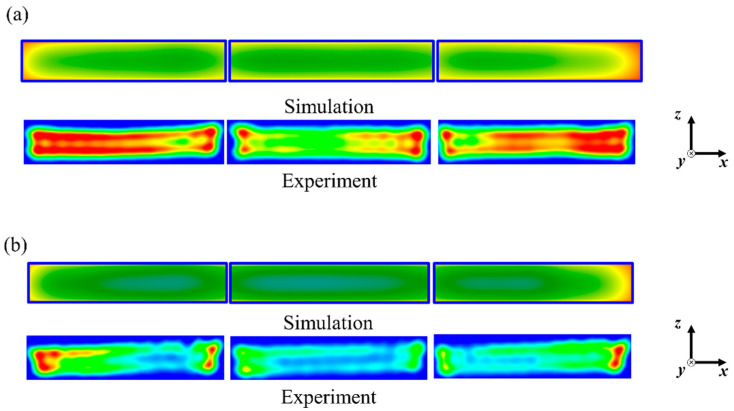
Comparisons of heating patterns on vertically cut sections of model foods: (**a**) 0.0%-added-salt and (**b**) 0.7%-added-salt model foods.

The effect of decreased penetration depth due to added salt content on heating patterns is shown in Figure 7 with the heating patterns of vertically cut sections (on the *x–z* plane). Both the 0.0%- and 0.7%-salt-added model foods showed an edge heating effect during microwave heating, although the color intensity was different between the simulation and experiment results. The heating patterns would have been induced by both the surface heating from the hot water and the electric field distribution in the microwave cavity. Distinguished anti-nodes were presented at the surface of the model food in the simulation results (Figure 3b). Regardless of the cavity width, consistent heating patterns were presented between the simulation and the experimental results, also implying that the design simplifications of the microwave horns used for the simulation calculations were not critical to the projections of the electric field distribution. Based on these results, improvements to heating uniformity could be developed from appropriate changes in the phase shifts and cavity dimensions, with the predicted and actual effects being determined for various food products using model foods of matched dielectric properties.

## 4. Conclusions

This was the first assessment to determine the electric fields and heating patterns of a MATS-B-based system by a computer simulation model, for better understanding of the effects of the cavity dimensions and food carrier on the heating uniformity in food trays. A MATS system set to a 180° phase difference between the top and bottom microwaves is predicted to cause more surface heating than a system set to a 0° phase difference. Experimental validation of the computer simulation model against the Maillard browning heating patterns of model food in the trays showed alignment of the predicted heating patterns with those observed on the trays. The computer simulation in this research will provide a useful approach to improving the uniformity of heating for food products in the MATS sterilization system. With greater computer processing power, it can also be used as a predictive tool for the design optimization of full-scale MATS manufacturing systems. Thermal analysis of actual temperature changes will be required in future work.

## Figures and Tables

**Table 1:** Simulation settings for the microwave-assisted thermal sterilization (MATS) system.

		Parameter	Set Value
Process conditions		Time step (s)	12
		Exciting mode of port	TE 10
		Excitation amplitude per port (V/m)	46.54
		Initial temperature of food (°C)	85
		Initial temperature of carrier (°C)	85
		Temperature of water in heating cavity (°C)	121
		Moving speed (cm/min)	168
Properties of materials ^1^	PTFE plastic	Dielectric constant	2.02
		Loss factor	7.86 × 10^−4^
		Density (kg/m^3^)	1400
		Specific heat (J/(kg·°C))	1300
		Thermal conductivity (W/(m·°C))	0.447
	Model food	Volumetric specific heat (MJ/(m^3^·°C))	3.11/3.89
	(0.0/0.7% salt)	Thermal conductivity (W/(m·°C))	0.852/0.773
	Water	Dielectric constant	50.0
		Loss factor	0.76
		Density (kg/m^3^)	950
		Specific heat (J/(kg·°C))	4200
		Thermal conductivity (W/(m·°C))	0.677

^1^ Average material properties obtained in the range of sterilization processing of MATS.

**Table 2:** Process parameter settings for MATS-B experimental validation.

Section	Process Condition		Set Value
Pre-heating	Temperature [°C]		85
	Time [min]		25
Microwave heating	First pass	Temperature [°C]	110
		Generator power [kW]	9
		Dwell time [min] ^1^	0.1
	Second pass	Temperature [°C]	115
		Generator power [kW]	9
		Dwell time [min]	0.36
	Third pass	Temperature [°C]	121
		Generator power [kW]	8
		Dwell time ^1^ [min]	0.1
	Fourth pass	Temperature [°C]	125
		Generator power [kW]	8
		Dwell time [min]	0.1
Holding	Temperature [°C]		125
	Time [min]		5
Cooling	Temperature [°C]		30/15
	Time [min]		5/5

^1^ Dwell time is defined as the holding time between the food transport carrier totally passing the microwave horn applicator and the commencement of the next microwave heating application.

## Data Availability

Data is contained within the article.

## References

[1] Tang J. (2015). Unlocking Potentials of Microwaves for Food Safety and Quality. J. Food Sci..

[2] Chizoba Ekezie F.-G., Sun D.-W., Han Z., Cheng J.-H. (2017). Microwave-assisted food processing technologies for enhancing product quality and process efficiency: A review of recent developments. Trends Food Sci. Tech..

[3] Tang J., Hong Y.-K., Inanoglu S., Liu F. (2018). Microwave Pasteurization for Ready-to-Eat Meals. Curr. Opin. Food Sci..

[4] Chandrasekaran S., Ramanathan S., Basak T. (2012). Microwave material processing—A review. AIChE J..

[5] Raaholt B.W., Isaksson S. (2017). Improving the heating uniformity in microwave processing. The Microwave Processing of Foods.

[6] Zhu H., He J., Hong T., Yang Q., Wu Y., Yang Y., Huang K. (2018). A rotary radiation structure for microwave heating uniformity improvement. Appl. Therm. Eng..

[7] Luan D., Tang J., Pedrow P.D., Liu F., Tang Z. (2016). Analysis of electric field distribution within a microwave assisted thermal sterilization (MATS) system by computer simulation. J. Food Eng..

[8] Jain D., Tang J., Liu F., Tang Z., Pedrow P.D. (2018). Computational evaluation of food carrier designs to improve heating uniformity in microwave assisted thermal pasteurization. Innov. Food Sci. Emerg..

[9] Luan D., Tang J., Pedrow P.D., Liu F., Tang Z. (2013). Using mobile metallic temperature sensors in continuous microwave assisted sterilization (MATS) systems. J. Food Eng..

[10] Resurreccion F.P., Luan D., Tang J., Liu F., Tang Z., Pedrow P.D., Cavalieri R. (2015). Effect of changes in microwave frequency on heating patterns of foods in a microwave assisted thermal sterilization system. J. Food Eng..

[11] Chen H., Tang J., Liu F. (2008). Simulation model for moving food packages in microwave heating processes using conformal FDTD method. J. Food Eng..

[12] WSU Insider India Partnership Deploys New WSU Microwave Technologies for Safer Meals. https://news.wsu.edu/2020/03/31/india-partnership-deploys-new-wsu-microwave-technologies-safer-meals/.

[13] Pathak S., Liu F., Tang J. (2003). Finite difference time domain (FDTD) characterization of a single mode applicator. J. Microwave Power Electromagn. Energy.

[14] Resurreccion F.P., Tang J., Pedrow P., Cavalieri R., Liu F., Tang Z. (2013). Development of a computer simulation model for processing food in a microwave assisted thermal sterilization (MATS) system. J. Food Eng..

[15] Zhao X., Yan L., Huang K., Grundas S. (2011). Review of Numerical simulation of Microwave Heating Process. Advances in Induction and Microwave Heating of Mineral and Organic Materials.

[16] Jain D., Tang J., Pedrow P.D., Tang Z., Sablani S., Hong Y.-K. (2019). Effect of changes in salt content and food thickness on electromagnetic heating of rice, mashed potatoes and peas in 915 MHz single mode microwave cavity. Food Res. Int..

[17] Balanis C.A. (1999). Advanced Engineering Electromagnetics.

[18] Datta A.K., Datta A.K. (2001). Fundamentals of Heat and Moisture Transport for Microwaveable Food Product and Process Development. Handbook of Microwave Technology for Food Application.

[19] Geankoplis C.J. (2003). Transport Processes and Separation Process Principles:(Includes Unit Operations).

[20] Ehrlich P. (1953). Dielectric properties of teflon from room temperature to 314 °C and from frequencies of 102 to 105 c/s. J. Res. Nat. Bur. Stand..

[21] Ghodgaonkar D.K., Varadan V.V., Varadan V.K. (1989). A free-space method for measurement of dielectric constants and loss tangents at microwave frequencies. IEEE Trans. Instrum. Meas..

[22] Rattanadecho P. (2006). The simulation of microwave heating of wood using a rectangular wave guide: Influence of frequency and sample size. Chem. Eng. Sci..

[23] Luan D., Tang J., Pedrow P.D., Liu F., Tang Z. (2015). Performance of mobile metallic temperature sensors in high power microwave heating systems. J. Food Eng..

[24] Gupta R., Mikhaylenko G., Balasubramaniam V.M., Tang J. (2011). Combined pressure–temperature effects on the chemical marker (4-hydroxy-5-methyl- 3(2H)-furanone) formation in whey protein gels. LWT Food Sci. Technol..

[25] Wang Y., Tang J., Rasco B., Wang S., Alshami A.A., Kong F. (2009). Using whey protein gel as a model food to study dielectric heating properties of salmon (Oncorhynchus gorbuscha) fillets. LWT Food Sci. Technol..

[26] Wang Y., Lau M.H., Tang J., Mao R. (2004). Kinetics of chemical marker M-1 formation in whey protein gels for developing sterilization processes based on dielectric heating. J. Food Eng..

[27] Auksornsri T., Tang J., Tang Z., Lin H., Songsermpong S. (2018). Dielectric properties of rice model food systems relevant to microwave sterilization process. Innov. Food Sci. Emerg..

[28] Pandit R.B., Tang J., Liu F., Mikhaylenko G. (2007). A computer vision method to locate cold spots in foods in microwave sterilization processes. Pattern Recogn..

[29] Soto-Reyes N., Temis-Pérez A.L., López-Malo A., Rojas-Laguna R., Sosa-Morales M.E. (2015). Effects of Shape and Size of Agar Gels on Heating Uniformity During Pulsed Microwave Treatment. J. Food Sci..

[30] Tang Z., Hong T., Liao Y., Chen F., Ye J., Zhu H., Huang K. (2018). Frequency-selected method to improve microwave heating performance. Appl. Therm. Eng..

[31] Plaza-Gonzalez P., Monzo-Cabrera J., Catala-Civera J.M., Sanchez-Hernandez D. (2005). Effect of mode-stirrer configurations on dielectric heating performance in multi-mode microwave applicators. IEEE Trans. Microw. Theory Tech..

[32] Bornhorst E.R., Tang J., Sablani S.S., Barbosa-Cánovas G.V. (2017). Development of model food systems for thermal pasteurization applications based on Maillard reaction products. LWT Food Sci. Technol..

[33] Bornhorst E.R., Tang J., Sablani S.S., Barbosa-Cánovas G.V., Liu F. (2017). Green pea and garlic puree model food development for thermal pasteurization process quality evaluation. J. Food Sci..

[34] Zhang W., Tang J., Liu F., Bohnet S., Tang Z. (2014). Chemical marker M2 (4-hydroxy-5-methyl-3(2H)-furanone) formation in egg white gel model for heating pattern determination of microwave-assisted pasteurization processing. J. Food Eng..

[35] Auksornsri T., Bornhorst E.R., Tang J., Tang Z., Songsermpong S. (2018). Developing model food systems with rice based products for microwave assisted thermal sterilization. LWT Food Sci. Technol..

[36] Pandit R.B., Tang J., Mikhaylenko G., Liu F. (2006). Kinetics of chemical marker M-2 formation in mashed potato—a tool to locate cold spots under microwave sterilization. J. Food Eng..

[37] Wang R., Zhang M., Mujumdar A.S., Jiang H. (2011). Effect of salt and sucrose content on dielectric properties and microwave freeze drying behavior of re-structured potato slices. J. Food Eng..

[38] Wang J., Tang J., Park J.W., Rasco B., Tang Z., Qu Z. (2019). Thermal gelation of Pacific whiting surimi in microwave assisted pasteurization. J. Food Eng..

[39] Hong Y.-K., Liu F., Tang Z., Pedrow P.D., Sablani S.S., Yang R., Tang J. (2021). A simplified approach to assist process development for microwave assisted pasteurization of packaged food products. Innov. Food Sci. Emerg..

